# Modulation of the Gut Microbiota by Krill Oil in Mice Fed a High-Sugar High-Fat Diet

**DOI:** 10.3389/fmicb.2017.00905

**Published:** 2017-05-17

**Authors:** Chenyang Lu, Tingting Sun, Yanyan Li, Dijun Zhang, Jun Zhou, Xiurong Su

**Affiliations:** ^1^School of Marine Science, Ningbo UniversityNingbo City, China; ^2^Department of Food Science, Cornell UniversityNew York, NY, United States

**Keywords:** krill oil, hyperlipidemia, gut microbiota, dose-dependent, mouse

## Abstract

Multiple lines of evidence suggest that the gut microbiota plays vital roles in metabolic diseases such as hyperlipidemia. Previous studies have confirmed that krill oil can alleviate hyperlipidemia, but the underlying mechanism remains unclear. To discern whether krill oil changes the structure of the gut microbiota during the hyperlipidemia treatment, 72 mice were acclimatized with a standard chow diet for 2 weeks and then randomly allocated to receive a standard chow diet (control group, *n* = 12) or a high-sugar-high-fat (HSHF) diet supplemented with a low (100 μg/g·d, HSHF+LD group, *n* = 12), moderate (200 μg/g·d, HSHF+MD group, *n* = 12) or high dosage of krill oil (600 μg/g·d, HSHF+HD group, *n* = 12), simvastatin (HSHF+S group, *n* = 12) or saline (HSHF group, *n* = 12) continuously for 12 weeks. The resulting weight gains were attenuated, the liver index and the low-density lipoprotein, total cholesterol and triglyceride concentrations showed a stepwise reduction in the treated groups compared with those of the control group. A dose-dependent modulation of the gut microbiota was observed after treatment with krill oil. Low- and moderate- doses of krill oil increased the similarity between the composition of the HSHF diet-induced gut microbiota and that of the control, whereas the mice fed the high-dose exhibited a unique gut microbiota structure that was different from that of the control and HSHF groups. Sixty-five key operational taxonomic units (OTUs) that responded to the krill oil treatment were identified using redundancy analysis, of which 26 OTUs were increased and 39 OTUs were decreased compared with those of the HSHF group. In conclusion, the results obtained in this study suggest that the structural alterations in the gut microbiota induced by krill oil treatment were dose-dependent and associated with the alleviation of hyperlipidemia. Additionally, the high-dose krill oil treatment showed combined effects on the alleviation of hyperlipidemia and obesity.

## Introduction

Humans face various metabolic diseases, including obesity, hyperlipidemia, hyperglycemia, and hypertension (Eckel et al., 2005). Hyperlipidemia comprises a group of plasma lipoprotein abnormalities, including increased triglycerides (TG), total cholesterol (TC), low density lipoprotein cholesterol (LDL), and reduced high density lipoprotein cholesterol (HDL) concentrations (Chen et al., 2014b). Hyperlipidemia is a dominant risk factor that contributes to cardiovascular disease (Abliz et al., 2014; Kim et al., 2015), and its development has become a difficult public health problem (Chen et al., 2014b). At present, drug therapy is the preferred treatment for hyperlipidemia. Various drugs have been used clinically, including simvastatin (Farnier and Davignon, 1998), acipimox (Shih et al., 1997), and clofibrate (Grundy et al., 1972). Generally, drug therapy is not regarded as an optimal long-term therapy due to its relatively high costs and adverse side effects. Therefore, intervention strategies are needed for the treatment of hyperlipidemia.

Accumulating evidence suggests that a disrupted balance between the host and the gut microbiota plays a vital role in lipid metabolism and the development of hyperlipidemia (Ley et al., 2006; Turnbaugh et al., 2006). Probiotic feeding reduced the development of a fat-mass in mice fed a high-fat diet, with a corresponding decrease in the phylum Firmicutes, increase in the phylum Bacteroidetes, and changes in 102 distinct taxa in the gut microbiota (Everard et al., 2011). In the hyperlipidemic mouse model, *Lactobacillus* administration led to a significant reduction in the serum lipids, an increased abundance of phylum Bacteroidetes and Verrucomicrobia and a reduced proportion of the phylum Firmicutes (Chen et al., 2014a). In the zebrafish model, *Lactobacillus rhamnosus* decreased the lipid content by increasing the ratio of Firmicutes, decreasing the abundance of Actinobacteria, and down-regulating the genes involved in cholesterol and triglycerides metabolism (Falcinelli et al., 2015). Furthermore, the addition of green tea powder promoted the growth of *Lactobacillus* in the intestine and strongly decreased the hepatic triacylglycerol and cholesterol accumulation and the amount of *Akkermansia* in the small intestine in mice fed a high fat diet (Axling et al., 2012). Some Chinese medicines have shown positive effects on hyperlipidemia control via modulation of the gut microbiota. The abundance of *Sporobacter termitidis* and *Akkermansia muciniphila* were increased significantly, whereas the proportions of *Escherichia coli* and *Parabacteroides distasonis* were reduced after treatment of hyperlipidemic B6 mice with *Rhizoma coptidis* alkaloids (He et al., 2016). The berberine supplement enriched short chain fatty acid (SCFA)—producing bacteria and reduced microbial diversity, which might contribute to the beneficial effects on dyslipidemia and hypercholesterolemia treatment (Kong et al., 2004; Zhang et al., 2015). In conclusion, the changes in the gut microbiota play an important role in the regulation of lipid metabolism and the alleviation of hyperlipidemia, and may have potential as a new hyperlipidemia treatment strategy.

Krill oil is extracted from *Euphausia superb* and contains eicosapentaenoic acid (EPA) and docosahexaenoic acid (DHA; Maki et al., 2009). The fatty acids in krill oil are present as phospholipids, which are absorbed well by the intestine compared with other triglycerides oils of marine origin (Harris et al., 1988; Araujo et al., 2014). Previous studies suggested that krill oil supplementation alleviated hyperlipidemia. For example, krill oil treatment suppressed lipid synthesis, up-regulated the genes involved in lipid oxidation, and down-regulated genes in lipogenesis (Burri et al., 2011; Ferramosca et al., 2012). Krill oil supplementation reduced the TG and TC levels and alleviated the hepatomegaly and hepatic steatosis induced by a high-fat diet (Tandy et al., 2009). Another study found that the hyperplasia and total histology score were dramatically decreased and that the serum cytokines levels remained unchanged after krill oil treatment (Ierna et al., 2010). In addition to a high concentration of omega-3 fatty acids, krill oil also contains astaxanthin, which is responsible for the deep red color (Figure S1). Astaxanthin is a strong lipid-soluble antioxidant that not only protects krill oil from oxidation (Hussein et al., 2007; Ikeuchi et al., 2007) but also provides additional health-promoting properties related to inflammation and hyperlipidemia (Bunea et al., 2004; Deutsch, 2007). At present, we have confirmed the benefits of krill oil in human health. However, there is a lack of evidence concerning whether krill oil alleviates hyperlipidemia via modulation of the gut microbiotan, and the dose-dependent effects of krill oil on hyperlipidemia remains unclear.

In this study, a randomized approach was used to assess the effect of krill oil on hyperlipidemia in mice. The biochemical blood indices, liver index, and shift in the structure of the gut microbiota in response to krill oil treatment at different doses were examined in mice. Our results provide new insights into the roles of the gut microbiota following treatment with different dose of krill oil for hyperlipidemia. We propose that this study will aid in the screening of intervention strategies for the amelioration of hyperlipidemia.

## Materials and methods

### Experimental design

The experimental and animal care procedures were performed in accordance with the guidelines prepared by the Ningbo University Laboratory Animal Center (affiliated with the Zhejiang Laboratory Animal Common Service Platform, Ningbo, China). All protocols were approved by the Ningbo University Laboratory Animal Center under permit number SYXK (ZHE 2008-0110).

Seventy-two 10-week-old ICR mice (22.1 ± 2.6 g, male, purchased from Laboratory Animal Center of Zhejiang province, SCXK (Zhejiang) 2014-0001, No. 1605200003) were randomly divided into six groups (12 mice per group) after 2 weeks of acclimatization with a standard chow diet. The twelve mice in each group were divided into three cages (four mice per cage). The mice in the control group were fed a standard chow diet (purchased from the Laboratory Animal Center of Ningbo University, Ningbo, China). The mice in the high-sugar high-fat (HSHF) diet group were fed a HSHF diet (20% lard, 20% sucrose, 2.5% cholesterol, 1% cholic acid salt, and 56.5% standard chow diet). These two groups simultaneously received saline by gavage. The mice in the HSHF+S group were maintained on the HSHF diet with Shujiangzhi (simvastatin, Merck & Co., Inc, Hangzhou, China) administration by gavage at a dosage of 1 μg/g·d for 12 weeks. The other three groups were maintained on the HSHF diet with a gavage of krill oil (purchased from Sino-Ocean Co., Ltd., Dalian, China) by gavage at low (100 μg/g·d, HSHF+LD group), moderate (200 μg/g·d, HSHF+MD group), and high dosages (600 μg/g·d, HSHF+HD group) for 12 weeks. The mice were kept in the same house during the experiments, and none of the mice died.

The body weights and food intake were measured weekly. After 12 weeks, fecal samples were collected again from each cage (representing four mice). Blood samples were collected from each mouse and immediately stored at −80°C. Organs from each mouse, including the brain, kidney, heart, liver, spleen, and lung, were excised and weighed. A viscera index was calculated using the formula: organ weight/body weight (mg/g).

### Measurement of blood biochemical indices

The TC, TG, HDL, and LDL levels in the blood samples were measured using kits from the Jiancheng Bioengineering Institute (Nanjing, China).

### Measurement of the krill oil component

The fatty acid composition was measured via chromatography-mass spectrometry (GC-MS) as previously described (Satil et al., 2003). An Agilent 7890/M7-80EI system with a VOCOL column (60 m × 0.32 mm) was used in this study, and helium was used as the carrier gas. The GC oven temperature began at 60°C, increased to 260°C at a rate of 5°C/min, and then was maintained at 260°C for 40 min. The gas flow rate was 50 mL/min. The temperature of the injector was maintained at 260°C. The detected mass ranged from 30 to 425 m/z.

The astaxanthin concentration in the krill oil was measured via high-performance liquid chromatography (HPLC) as previously described (López-Cervantes et al., 2006). The HPLC was operated at a rate of 1 mL/min with an SS Exil ODS column (250 × 4.6 mm, 5 μm) and detected at the 476 nm wavelength. A mixture of water, methanol, dichloromethane, and acetonitrile at a 4.5:28:22:45.5 ratio (v/v/v/v) was used as the mobile phase. An astaxanthin standard was purchased from Sigma-Aldrich Co. LLC (St. Louis, MO, USA).

### Total DNA extraction, PCR, and sequencing

Total DNA was isolated from each sample (feces from the same cage) using a previously described method as Yu and Morrison (2004). The quantity of the extracted DNA was measured using a Thermo NanoDrop 2000C (Thermo Fisher Scientific, Waltham, MA, USA).

The PCR primers were designed based on the sequence of the V3 and V4 hypervariable regions of the bacterial 16S rRNA gene. The primers 319F 5′-ACTCCTACGGGAGGCAGCAG-3′ and 806R 5′-GGACTACHVGGGTWTCTAAT-3′ were designed with a barcode sequence that was unique to each sample. Amplification reactions were performed in 25 μL volume containing 12.5 μL Premix Ex TaqTM Hot Start Version (Takara Biotechnology Co. Ltd, Dalian, China), 0.1 μM of each primer, and 20 ng of template. Amplification was initiated at 98°C for 30 s, followed by 35 cycles of denaturation at 98°C for 10 s, primer annealing at 54°C for 30 s, extension at 72°C for 45 s, and final extension for 10 min. PCR reactions for each sample were performed with a negative control in each run. The presence of amplicons was confirmed using gel electrophoresis, and the PCR products were normalized using AxyPrepTM Mag PCR Normalizer and then sequenced using the MiSeq system constructed with the Illumina Nextera XT Index kit in Sangon Biotech Co., Ltd. (Shanghai, China). Sequencing was performed on an Illumina MiSeq (Illumina, San Diego, CA) using 2 × 300 bp paired-end sequencing and multiple sequencing runs in accordance with the manufacturer's instructions.

### Data analysis and statistical analysis

Raw FASTQ files were multiplexed and filtered using QIIME (version 1.8.0) (Caporaso et al., 2010) according to the following steps: (1) the 300-bp reads were truncated at any position when an average quality score <20 on a 50-bp sliding window was observed, and truncated reads shorter than 50-bp were removed; (2) data belonging to each sample were identified via the barcode sequence, and reads containing undetected nucleotides (N) or two-nucleotide primer mismatches were removed; and (3) sequences that overlapped more than 10-bp were assembled, and the reads that could not be assembled were discarded. Operational taxonomic units (OTUs) were clustered using Usearch (version 7.1) (Edgar, 2010) at a similarity level of 97%. The most abundant sequence of each OTU in all the samples was selected as the representative sequence. The RDP classifier (Wang et al., 2007) was used to annotate the representative OTU sequences, and taxonomic information was obtained for each OTU. The phylogenetic tree and the normalized relative abundance of OTUs were used for weighted and unweighted UniFrac principal coordinate analysis (PCoA) via vegan package in R software (version 3.2). The community richness index and community diversity were calculated using Mothur (version 1.30.1) and the reads number normalized to the same number (smallest) to reduce biases in sequencing depth for alpha and beta diversity analysis (Schloss et al., 2009).

All data are shown as the means ± *S.D*. The ANOVA test and Tukey's *post-hoc* test (SPSS, version 19.0, Chicago, IL, USA) were used to analyse data with a normal distribution, and the Mann-Whitney test (MATLAB R2012a, Natick, MA, USA) was used to analyse data that did not meet the assumptions of the ANOVA. *P* < 0.05 was defined as the standard criterion for statistical significance. Redundancy analysis models were constructed to identify specific bacterial phylotypes whose abundance was changed by HSHF feeding and by krill oil treatment. The relative abundance of each OTU was normalized and log-transformed, and used to construct RDA models to find the OTUs that were different among groups with Canoco for Windows 4.5 (Microcomputer Power, Ithaca, NY, USA) according to the manufacturer's instructions, and the type of treatment and dose of krill oil were used as environmental variables. Responding OTUs that explained more than 4% of the variability of the samples selected. Statistical significance was assessed by MCPP with 499 random permutations under the full model. Correlations between gut microbial composition (OTU abundances) and the average of the host parameters of hyperlipidemia in the same cage were identified using Spearman's correlation (SPSS, version 19.0, Chicago, IL, USA). Correlations between gut microbial composition and the average of the host parameters in the same cage were considered significant when *P* < 0.05. False discovery rate control was used to account for multiple comparisons when evaluating correlations between OTUs and host parameters in SPSS software, and correlations were deemed significant at false discovery rate < 0.25.

### Accession number

The sequences have been deposited in the NCBI Sequence Read Archive Database under accession number SRP095937.

## Results

### The major components of krill oil

The krill oil purchased from Sino-Ocean Co., Ltd. (Dalian, China; shown in Figure S1) was a transparent, dark red, and viscous oil. The krill oil contained 30.04 wt % unsaturated fatty acids. The DHA and EPA concentrations were 2.62 wt % and 5.56 wt %, respectively. Eighty percent of the total fatty acids in the krill oil were phospholipids (568 mg/g; Table S1). The astaxanthin concentration was 37.43 mg/100 g.

### Krill oil attenuates features of hyperlipidemia in HSHF diet fed mice

**Figure 1:**
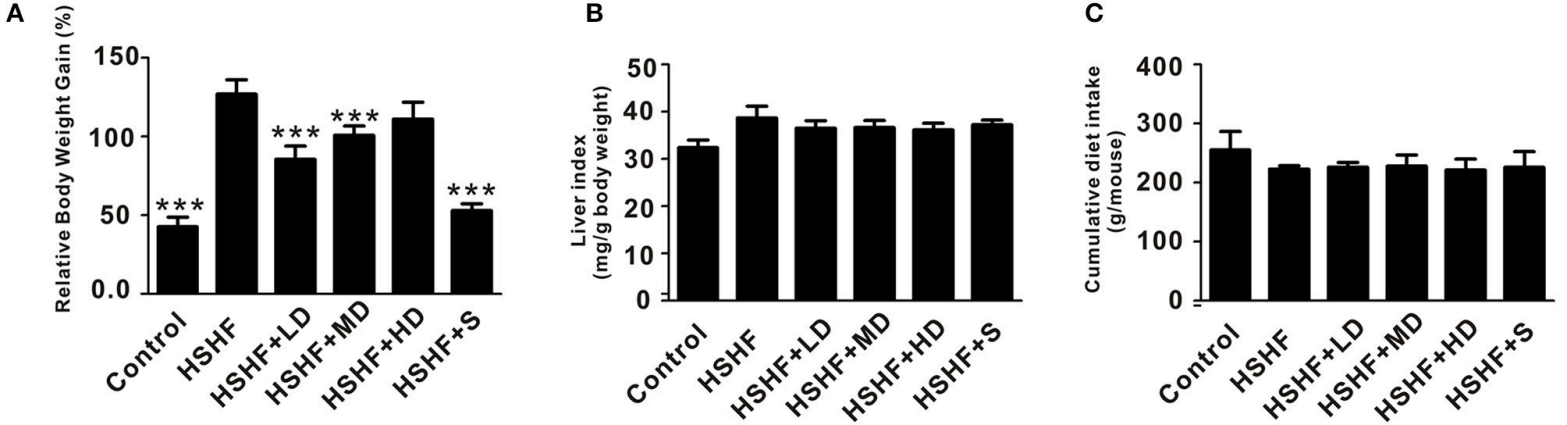
**Effects of krill oil treatment on the physical features of mice fed the HSHF diet. (A)** The relative body weight gain of HSHF diet fed mice with krill oil supplementation. **(B)** Liver index of HSHF diet fed mice with krill oil supplementation. **(C)** Total food intake of HSHF diet fed mice with krill oil supplementation. TC, total cholesterol. TG, triglyceride. HDL, high-density lipoprotein. LDL, low-density lipoprotein. Data are presented as the means ± *S.D*. Differences were assessed by ANOVA. ^***^P < 0.001 vs. HSHF group.

**Figure 2:**
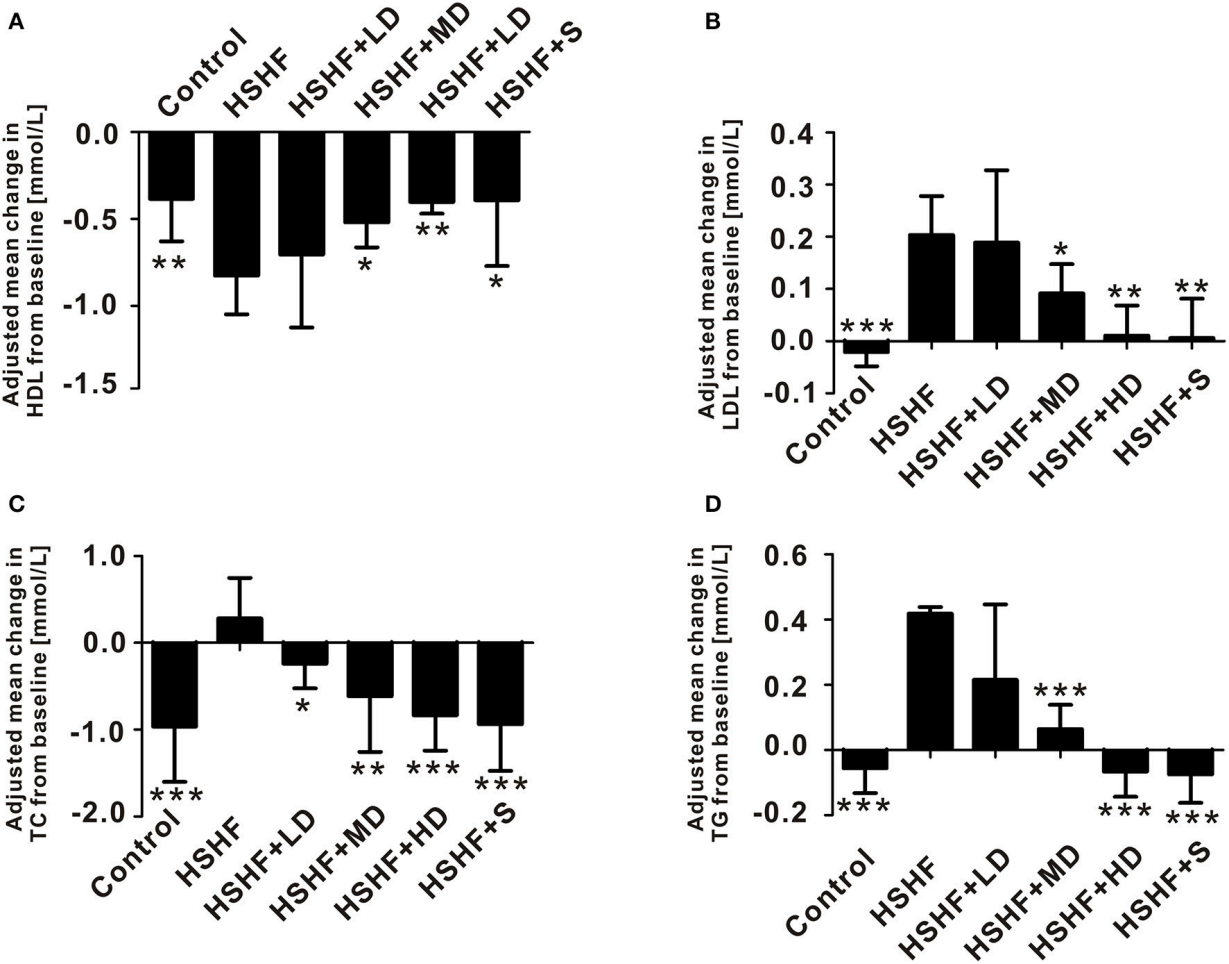
**Krill oil significantly improved serum lipid control in mice fed the HSHF diet. (A)** Change in the HDL level. **(B)** Change in the LDL level. **(C)** Change in the TC level. **(D)** Change in the TG level. Data are presented as the means ± *S.D*. Differences were assessed by ANOVA. ^*^P < 0.05, ^**^P < 0.01, and ^***^P < 0.001 vs. HSHF group.

The mice in the HSHF group gained more weight than the mice in the control group (*P* < 0.001; Figure 1A, Table S2). The mice in the HSHF group developed the hallmark features of hyperlipidemia, with a significant increase in the LDL, TC, and TG concentrations (*P* < 0.001) and a significant decrease in the HDL concentration (*P* < 0.01; Figure 2). Additionally, the mice in the HSHF group experienced a significant increase in their liver weights, the liver index increased by 19.4% (*P* < 0.001), and the other visceral indices remained unchanged (Figure 1B, Figure S2).

Twelve weeks of dietary supplementation with different doses of krill oil significantly attenuated the HSHF diet-induced weight gain (Figure 1A, Table S2), although no differences were observed in the food intake (Figure 1C). Furthermore, krill oil treatment significantly attenuated hyperlipidemia in the mice fed the HSHF diet. The LDL, TC, and TG levels were significantly decreased in the HSHF+MD group (*P* < 0.05) and the HSHF+HD group (*P* < 0.01) compared to those in the HSHF group. The HSHF+LD group showed a significant reduction in the TC level (*P* < 0.05; Figure 2, Table S3). Krill oil supplementation decreased the liver index of all groups compared to that of the HSHF group, with reduction of 5.6, 5.3, and 6.6% (*P* > 0.05) in the HSHF+LD, HSHF+MD, and HSHF+HD groups, respectively. The other visceral indices showed no response to krill oil supplementation at any of the doses (Figure 1B, Figure S2). The HSHF+S group showed the same pattern for the biochemical indices and liver index as the HSHF+HD group. Altogether, these results demonstrated the dose-dependent effect and safety of krill oil in mitigating HSHF diet induced weight gain and hyperlipidemia symptoms.

### The HSHF diet and krill oil treatment lead to changes in the gut microbiota structure

To elucidate the contributions of the krill oil treatment to the gut microbiota, we collected fecal samples from 72 mice after 12 weeks of treatment and performed a high-throughput sequencing analysis of the V3 and V4 hypervariable regions of the bacterial 16S rRNA gene. And the raw reads in each group are: control group (39,207, 36,383, and 26,214), HSHF group (40,795, 31,757, and 21,003), HSHF+LD group (21,404, 41,845, and 23,910), HSHF+MD group (37,709, 45,256, and 30,429), HSHF+HD group (27,117, 29,835, and 26,325), and HSHF+S group (34,849, 30,748, and 19,576).

The diversity of the microbial communities was measured using the Shannon and Simpson diversity indices (Figure S3). A significantly decreased Shannon index (*P* < 0.05) and a significantly increased Simpson index (*P* < 0.05) were measured in the HSHF group compared with those in the control group, which indicated that bacterial diversity was decreased after the HSHF diet treatment. The krill oil treatment restored the bacterial diversity regardless of the krill oil dose, with an increased Shannon index as well as a reduced Simpson index compared with those in the HSHF group.

**Figure 3:**
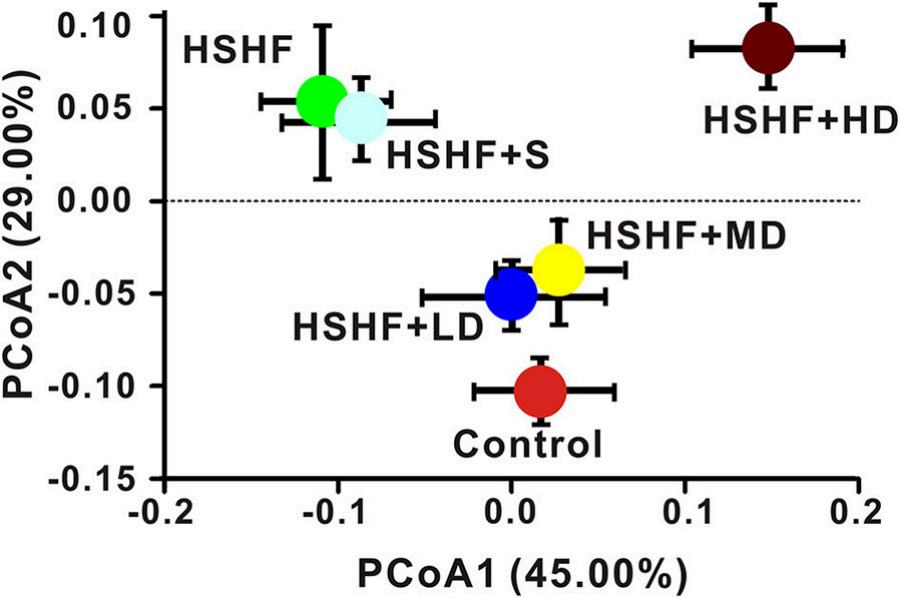
**Dose-dependent change in the composition of mice fed the HSHF diet and treated with different krill oil doses via Weighted Unifrac PCoA analysis**. Data are presented as the means ± *S.D*.

The overall compositions of the gut microbiota in the six groups were analyzed via Weighted and Unweighted UniFrac PCoA (Figure 3, Figure S4). The low- and moderate-dose krill oil treatments shifted the overall compositions of the gut microbiota in the HSHF group toward the composition of the control group, whereas the HSHF+HD group showed a unique pattern that was different from that in the HSHF and control groups. Interestingly, we identified a close relationship between the gut microbiota structures of the HSHF and HSHF+S, even though the LDL, TC, and TG indices were significantly reduced and the hyperlipidemia features were attenuated in the HSHF+S group. This finding indicated that the anti-hyperlipidemic activity of simvastatin was not caused by modulation of the gut microbiota.

### Microbial shifts in response to the krill oil treatment in mice fed the HSHF diet

**Figure 4:**
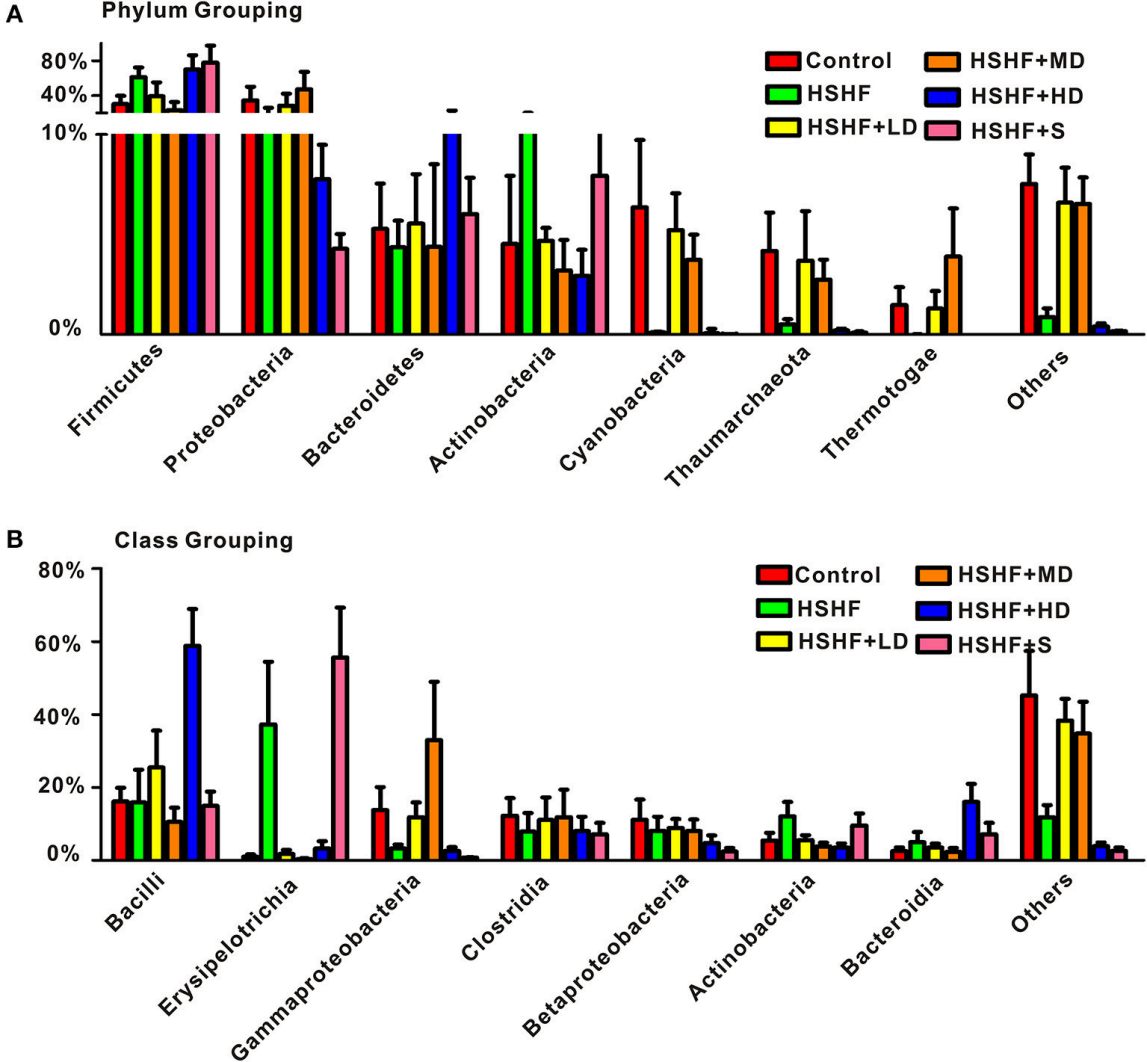
**RDP classifications of the average sequences reads. (A)** At the phylum level and **(B)** at the class level. Data are presented as the means ± *S.D*.

The RDP classifier clustered 98.93% of the reads into 15 phyla. The most abundant phyla in the control group were: Proteobacteria, Firmicutes, Cyanobacteria, Bacteroidetes, Actinobacteria, and Thaumarchaeota. After 12 weeks on HSHF diet, widespread changes in the structure of the gut microbiota were observed at the phylum level compared with that in the control group, with significant decreased proportions of Cyanobacteria (*P* < 0.05), Thaumarchaeota (*P* < 0.05), and significant increased proportions of Firmicutes (*P* < 0.05). The low and moderate-dose krill oil treatment attenuated the changes in the gut microbiota structures, whereas the high-dose treatment aggravated the changes in some phyla (Figure 4A, Table S4). However, the ratio of Firmicutes to Bacteroidetes increased after HSHF diet treatment (*P* < 0.01), whereas the krill oil treatment attenuated this increase (*P* < 0.01).

Bacilli, Erysipelotrichia, and Clostridia are common classes in phylum Firmicutes. The abundance of Erysipelotrichia significantly increased (*P* < 0.05) after the HSHF diet treatment, whereas the abundance of Bacilli and Clostridia decreased, although not reaching significant level. The extra addition of krill oil restored the abundance of these three classes regardless of the krill oil dose (Figure 4B, Table S5). The classes Gammaproteobacteria, Betaproteobacteria, and Alphaproteobacteria were the major classes in the Proteobacteria phylum. The abundances of these three classes increased in the HSHF+LD and HSHF+MD groups. However, the opposite change was observed in the HSHF+HD and HSHF+S groups (Figure 4B, Table S5).

A total of 385, 189, 311, 382, 163, and 173 genera were identified from the control, HSHF, HSHF+LD, HSHF+MD, HSHF+HD, and HSHF+S groups, respectively. Genera with >1% abundance in at least four groups were defined as abundant genera. Seven abundant genera were identified (*Lactobacillus, Bacillus, Streptococcus, Staphylococcus, Delftia, Ralstonia*, and *Serratia*; Table S6). The first four genera belong to phylum Firmicutes, and the latter three belong to phylum Proteobacteria. Among the seven abundant genera, only the detected abundance of *Ralstonia* increased after the HSHF diet treatment (*P* > 0.05), whereas the species in the genera *Streptococcus* and *Delftia* were significantly decreased (Table S6). Furthermore, the different doses of krill oil had various effects on the abundances of these seven genera. Detailed information is provided in Table S6.

### Key phylotypes respond to krill oil treatment in mice fed with HSHF diet

**Figure 5:**
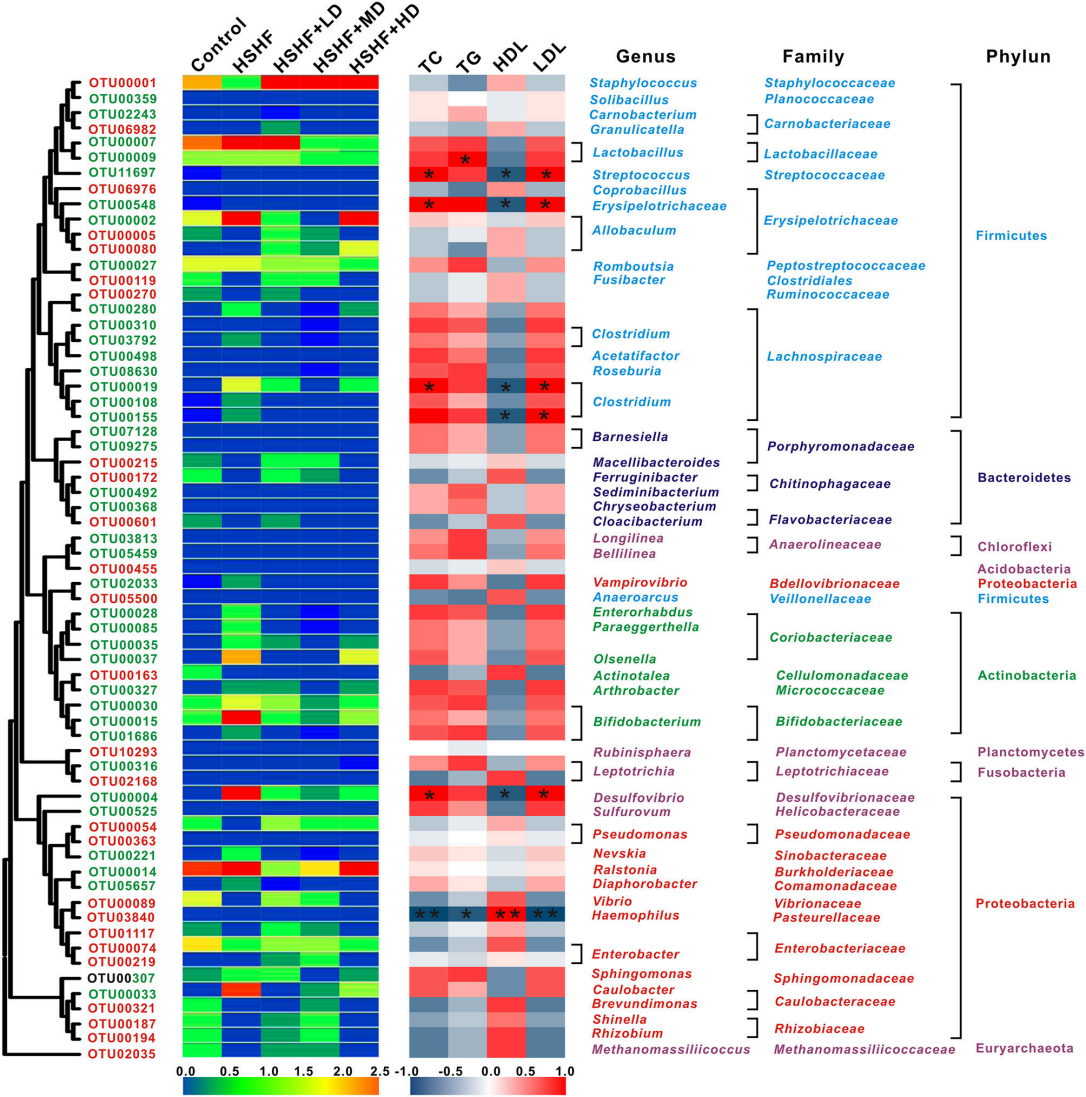
**Heat map of RDA-identified key OTUs responding to krill oil treatment in different doses and Spearman's correlation between the identified OTUs and the TC, TG, HDL, and LDL levels**. The color of the squares on the left indicate the average abundance of the OUT in each group. The OTUs are ordered via phylogenetic positions. The OTUs with a decreased abundance after HSHF diet treatment and a restored abundance after krill oil treatment are labeled in red, and the OTUs with an increased abundance after HSHF diet treatment and a restored abundance after krill oil treatment are labeled in green. The color of the squares in the right panel represents the *R*-value of Spearman's correlation between the OTU and the TC, TG, HDL, or LDL levels via SPSS. The phylum, family, and genus names of the OTUs are shown on the right. ^*^P < 0.05 and ^**^P < 0.01.

A total of 2,173, 1,352, 1,860, 2,064, 1,226, and 1,259 OTUs were identified in the control, HSHF, HSHF+LD, HSHF+MD, HSHF+HD, and HSHF+S groups, respectively. Because the anti-hyperlipidemic activity of simvastatin did not occur through modulation of the gut microbiota, further analysis was performed in these groups, except for the HSHF+S group. Sixty-five key OTUs that responded to the krill oil treatment were identified via redundancy analysis (Figure 5, Tables S7, S8). Among the 65 key OTUs, 26 OTUs were decreased by the HSHF diet and then increased by the krill oil treatment regardless of the dose, whereas the opposite pattern occurred for 39 OTUs. Among the 26 OTUs increased by krill oil, only OTU3840, which belongs to the *Haemophilus* genus, showed a significant negative correlation with the TC (*R* = −0.92, *P* < 0.01), TG (*R* = −0.88, *P* < 0.05) and LDL levels (*R* = −0.94, *P* < 0.01) and a significant positive correlation with the HDL level (*R* = 0.90, *P* < 0.01). *Four* OTUs (OTU80, OTU6976, OTU1, and OTU3840) from *Allobaculum, Coprobacillus, Staphylococcus*, and *Haemophilus*, respectively, showed the highest relative abundance after the high-dose krill oil (Figure 5, Tables S7, S8). Among the 39 OTUs that were decreased by krill oil treatment, 5 OTUs showed a significant positive correlation with the TC (*R* = 0.91, *P* < 0.05) and LDL (*R* = 0.90, *P* < 0.05) and a significant negative correlation with the HDL concentration (*R* = 0.93, *P* < 0.05). These OTUs belonged to *Erysipelotrichaceae* (*n* = 1), *Clostridium XlVa* (*n* = 2), *Streptococcus* (*n* = 1), and *Desulfovibrio* (*n* = 1). OTU9, which belongs to *Lactobacillus*, had a significant positive correlation only with the TG concentration (*R* = 0.92, *P* < 0.05). Seven OTUs (OTU492, OTU5459, OTU3813, OTU548, OTU9, OTU27, and OTU316) from *Sediminibacterium, Bellilinea, Longilinea, Erysipelotrichaceae, Lactobacillus, Romboutsia*, and *Leptotrichia*, respectively, showed the lowest relative abundance after HD krill oil treatment (Figure 5, Tables S7, S8).

## Discussion

To the best of our knowledge, this study is the first to elucidate the dose-dependent effect of krill oil on hyperlipidemia in a mouse model via a randomized design. In this study, the krill oil treatment provided significant and meaningful dose-dependent reductions in the physical (relative body weight gain and liver index; Figure 1, Table S2) and biochemical indices (LDL, TC, and TG; Figure 2) compared with those of the HSHF group. Additionally, the HDL level was significantly improved after krill oil treatment regardless of the dose. These findings were consistent with previous studies on dietary krill oil supplementation in mice fed a high-fat diet and in overweight humans (Maki et al., 2009; Tandy et al., 2009; Burri et al., 2011).

Krill oil supplementary in diet with low and moderate doses in this study maintained the structure of the gut microbiota like the control group, as well as the levels of HDL, LDL, TC, and TG. Previous studies have indicated that diet has a great influence (estimated at 57%, compared with 12% for genetic factors) on the structure of the gut microbiota (Tomasello et al., 2011). Consumption of a Western diet results in an increased abundance of *Bacteroides* spp, *Ruminococcus torques*, and *Bilophila wadsworthia* (Kamada et al., 2013), whereas the microbiota population with an Eastern diet has a higher abundance of *Prevotella* spp. (Ananthakrishnan, 2015). The improvement of vascular dysfunction by inulin-type fructans supplementary in the diet is linked to the increased nitric oxide producing bacteria, replenishment of abundance in *Akkermansia* and decreased abundance in bacterial taxa involved in secondary bile acid synthesis (Catry et al., 2017). The high consumption of fiber in the diet modified the gut microbiota populations and increased the abundance of acetate-producing bacteria (Marques et al., 2017).

However, the high-dose group, which exhibited further alleviation of the hyperlipidemia symptoms, showed a unique gut microbiota structure compared to that of the HSHF and control groups. Compared with the LD and MD groups, the HD group showed a decreased gut microbial diversity and the Proteobacteria abundance, and an increase in the Firmicutes abundance, although the Firmicutes to Bacteroidetes ratio was almost the same as the ratio in the MD group. The change in the gut microbiota structure in the HD group was similar to the previously “obesity model” (Marchesi et al., 2015; Alard et al., 2016; Denou et al., 2016). Krill oil and fish oil are mixtures of saturated and unsaturated fatty acids. In this study, a high concentration of supplementary fatty acids led to an increased body weight gain from 18.58 g in the LD group to 23.58 g in the HD group (Figure 1A). These results indicated that the krill oil treatment will lead to two effects in the same time: (1) the obesity and (2) the alleviation of hyperlipidemia. When krill oil intake at a low concentration (100 or 200 μg/g·d), the main effect focused on hyperlipidemia alleviation rather than obesity, and a control-like gut microbiota structure were observed in this study. When the concentration of krill oil increased to 600 μg/g·d, the effect on obesity was detected on increased body weight gain compared with the LD and MD groups, as well as the obesity-like structure with increased Firmicutes and decreased gut microbial diversity.

Seven abundant genera were detected in this study (Table S6), and the change in the abundance of these genera might explain the anti-hyperlipidemic effect. *Lactobacillus* and *Streptococcus*, which are lactic acid bacteria, were enriched by krill oil treatment in our study. The oral administration of *Lactobacillus* and *Streptococcus* successfully influenced the TC, TG, LDL, and HDL levels in addition to aminotransferase and aspartate aminotransferase activities in the rats with experimentally induced hyperlipidemia (Aderiye et al., 2007; Ngongang et al., 2016). The increased proportion of *Serratia* after krill oil treatment led to the accumulation of the compound FR177391, which had an anti-hyperlipidemic effect (Sato et al., 2005). In addition to hyperlipidemia, krill oil supplementation was also beneficial for the treatment of inflammatory and neurological disorders, and inflammatory disease was positively correlated with the *Ralstonia* and *Delftia* abundance (Tyler et al., 2013; Chen et al., 2014c; Bilgin et al., 2015; Tamburini and Clemente, 2016). In this study, krill oil treatment significantly decreased the proportion of *Ralstonia*, which might explain the anti-inflammatory effect. However, the reason for the unexpected increased abundance of *Delftia* remains unknown.

Among the 65 key OTUs that responded to krill oil treatment, 26 OTUs were increased and 39 were decreased, compared with those of the HSHF group, regardless of the krill oil dose (Figure 5). The OTUs that were increased in abundance after krill oil treatment, including the genera *Allobaculum* and *Enterobacter*, which were negatively correlated with hyperlipidemia phenotypes, and thus might represent potentially beneficial bacteria. The OTUs that showed reduced proportions, including the genera *Barnesiella, Clostridium XlVa, Desulfovibrionaceae*, and *Lactobacillus*, might include some harmful bacteria. Additionally, krill oil treatment led to the reduced abundance of *Desulfovibrionaceae* and *Clostridium XIVa* and increased proportion of *Allobaculum*. *Allobaculum* was shown to prevent dextran sulfate sodium-induced inflammation, whereas *Desulfovibrionaceae* and *Clostridium XIVa* that were involved in the obesity-related metabolic disorders or pro-inflammatory reactions (Zhang et al., 2010, 2012; Lam et al., 2012; Le Roy et al., 2013; Tuovinen et al., 2013).

However, among the OTUs detected both in the control and HSHF groups, 11 and 8 OTUs increased whereas 6 and 8 OTUs decreased after HSHF treatment, which belonged to *Bifidobacterium* probiotics and *Lactobacillus*, respectively. This finding indicated that many OTUs belonging to the same genus correlated in opposite directions. Previous studies have shown that different bacterial species in the same genus may respond differentially to the same environmental stressor, such as changing from a high-fat to a standard diet (Zhang et al., 2010, 2012). For instance, both *Bifidobacterium animalis* ssp. *lactis* GCL2505 and *B. longum* ssp. *longum* JCM1217T belong to genus *Bifidobacterium* but show different effects on the reduction of visceral fat accumulation, glucose tolerance improvement, and gut microbiota modulation (Aoki et al., 2017). Thus, identifying species-level phylotypes changes in response to treatment is important.

In conclusion, the results obtained in this study suggest that structural alterations in the gut microbiota induced by krill oil treatment are dose-dependent and associated with the alleviation of hyperlipidemia. Krill oil treatment will lead to the obesity and the alleviation of hyperlipidemia in the same time. The effects on the obesity was not obvious after low and moderate dose krill oil treatment, and a control-like gut microbiota structure with increased abundance of *Serratia, Delftia*, and *Streptococcus* in the gut and decreased proportion of *Ralstonia* were observed. Whereas with the increase of krill oil concentration, the effect on obesity was detected on increased body weight gain compared with the LD and MD groups, as well as the obesity-like gut microbiota structure with increased Firmicutes and decreased gut microbial diversity.

## Author contributions

CL and XS designed the study. CL, TS, YL, DZ, and JZ performed the experiments. CL, TS, YL, DZ, JZ, and XS provided reagents and materials. CL and TS analyzed the data. CL, TS, and XS wrote the main manuscript text and prepared the figures. All authors reviewed the manuscript.

## Funding

This work was supported by the Regional Demonstration Project of Marine Economic Innovation and Development (2013710), K. C. Wong Magna Fund in Ningbo University.

### Conflict of interest statement

The authors declare that the research was conducted in the absence of any commercial or financial relationships that could be construed as a potential conflict of interest.

## Supplementary material

Table S1 **The fatty acid components of the krill oil**.

Table S2 **The body weight of mice fed experimental diets**. Data are presented as the means ± *S.D*.

Table S3 **Serum lipid indices of mice fed experimental diets**. Data are presented as the means ± *S.D*. Differences were assessed by ANOVA. ^*^*P* < 0.05, ^**^*P* < 0.01, ^***^*P* < 0.001, compared with the HSHF group.

Table S4 **The RDP classifications of the sequence reads at the phylum level**. Data are presented as the means ± *S.D*. Differences were assessed by ANOVA. ^*^*P* < 0.05, compared with the HSHF group.

Table S5 **The RDP classifications of the sequence reads at the class level**. Data are presented as the means ± *S.D*. Differences were assessed by ANOVA. ^*^*P* < 0.05, compared with the HSHF group.

Table S6 **Seven abundant genera in all groups**. Data are presented as the means ± *S.D*. ^*^*P* < 0.05, compared with the HSHF group.

Table S7 **Sixty-five key OTUs respond to krill oil treatment as identified by redundancy analysis (RDA)**.

Table S8 **Relative average abundance of the 65 key OTUs responding to krill oil treatment as identified by redundancy analysis (RDA)**.

Figure S1 **Features of the krill oil. (A)** Concentrated krill oil. **(B)** Diluted krill oil.

Figure S2 **Viscera indices of the mice fed the HSHF diet with krill oil supplementation**. Data are presented as the means ± *S.D*.

Figure S3 **Different microbial diversity indices in different groups. (A)** ACE index. **(B)** Chao1 index. **(C)** Shannon index. **(D)** Simpson. ^*^*P* < 0.05 vs. control group.

Figure S4 **Dose-dependent change in the composition of mice fed the HSHF diet and treated with different krill oil doses via unweighted Unifrac PCoA analysis**. Data are presented as the means ± *S.D*.
