# Supplementary material for: Modulation of the Gut Microbiota by Krill Oil in Mice Fed a High-Sugar High-Fat Diet
Source: Front Microbiol. 2017 May 17;8:905. doi: 10.3389/fmicb.2017.00905 (PMC5434167; doi:10.3389/fmicb.2017.00905)
Supplement: Table S1 — The fatty acid components of the krill oil. [file Table1.PDF]

Table S1. The fatty acid components of the krill oil

NO.	Compound	Peak area (%)
1	Methyl tetradecanoate acid	11.43625
2	Pentadecanoic acid	1.107989
3	14-methyl-pentadecanoic acid	0.280595
4	Hexadecanoic acid	46.02376
5	9-hexadecenoic acid	2.904782
6	15-methyl-hexadecanoic acid	0.781221
7	Heptadecanoic acid	0.520586
8	16-methyl-heptadecanoic acid	0.924777
9	6,9,12,15-methyl-hexadecatetraenoate acid	0.200985
10	Methyl stearate acid	3.412002
11	9-Octadecenoic acid	7.97314
12	13-Octadecenoic acid	7.927584
13	11-Octadecenoic acid	0.393146
14	9,12-Octadecadienoic acid	1.159396
15	9,12,15-Octadecatrienoic acid	0.758249
16	Methyl stearidonate acid	1.047756
17	Methyl 9-eicosenoate acid	0.747246
18	EPA	5.555283
19	13-Docosenoic acid	1.656902
20	9,10,12-trimethoxy-octadecanoic acid	1.034113
21	Dodecanoic acid	0.696567
22	11-methoxy-octadecanoic acid	0.839415
23	DHA	2.618258
