# Supplementary material for: Modulation of the Gut Microbiota by Krill Oil in Mice Fed a High-Sugar High-Fat Diet
Source: Front Microbiol. 2017 May 17;8:905. doi: 10.3389/fmicb.2017.00905 (PMC5434167; doi:10.3389/fmicb.2017.00905)
Supplement: Table S2 — The body weight of mice fed experimental diets. Data are presented as the means ± S.D. [file Table2.PDF]

Table S2. The body weight of mice fed experimental diets. Data are presented as the means ± S.D.

	Standard diet	High-sugar-high-fat diet				
	Control	HSHF	HSHF+LD	HSHF+MD	HSHF+HD	HSHF+S
Initial BW (g)	24.10±0.56	21.03±1.04	21.82±1.51	21.35±1.53	21.42±2.51	24.30±1.63
Final BW(g)	34.37±1.28	47.66±1.60	40.41±1.73	42.83±2.63	44.99±3.19	37.15±2.58
BW gain (g)	10.27±1.37	26.63±1.27	18.58±1.13	21.59±1.45	23.58±0.94	12.85±1.30
