# Supplementary material for: Modulation of the Gut Microbiota by Krill Oil in Mice Fed a High-Sugar High-Fat Diet
Source: Front Microbiol. 2017 May 17;8:905. doi: 10.3389/fmicb.2017.00905 (PMC5434167; doi:10.3389/fmicb.2017.00905)
Supplement: Table S3 — Serum lipid indices of mice fed experimental diets. Data are presented as the means ± S.D. Differences were assessed by ANOVA. *P < 0.05, **P < 0.01, ***P < 0.001, compared with the HSHF group. [file Table3.PDF]

Table S3. Serum lipid indices of mice fed experimental diets. Data are presented as the means ± S.D. *P<0.05, **P<0.01, ***P<0.001, compared with the HSHF group.

	Normal diet	High-sugar-high-fat diet				
	Control	HSHF	HSHF+LD	HSHF+MD	HSHF+HD	HSHF+S
TC (mmol/L)	-0.96±0.64***	0.28±0.47	-0.24±0.29*	-0.61±0.64**	-0.83±0.41***	-0.94 ±0.54***
TG (mmol/L)	-0.06±0.08***	0.42±0.02	0.21±0.23	0.06±0.07***	-0.07±0.08***	-0.08±0.09***
HDL (mmol/L)	-0.38±0.24**	-0.83±0.22	-0.70±0.42	-0.52±0.14*	-0.40±0.07**	-0.39±0.38*
LDL (mmol/L)	-0.02±0.025***	0.20±0.074	0.19±0.14	0.09±0.06*	0.01±0.06**	0.01±0.08**
