# Supplementary material for: Modulation of the Gut Microbiota by Krill Oil in Mice Fed a High-Sugar High-Fat Diet
Source: Front Microbiol. 2017 May 17;8:905. doi: 10.3389/fmicb.2017.00905 (PMC5434167; doi:10.3389/fmicb.2017.00905)
Supplement: Table S4 — The RDP classifications of the sequence reads at the phylum level. Data are presented as the means ± S.D. Differences were assessed by ANOVA. *P < 0.05, compared with the HSHF group. [file Table4.PDF]

Table S4. **The RDP classifications of the sequence reads at the phylum level**. Data are presented as the means ± S.D. **P*<0.05, compared with the HSHF group.

PHYLUM	Control		HSHF	HSHF+LD		HSHF+MD		HSHF+HD		HSHF+S	
	Ratio (%)	Change [a]	Ratio (%)	Ratio (%)	Change [a]	Ratio (%)	Change [a]	Ratio (%)	Change [a]	Ratio (%)	Change [a]
Firmicutes	30.21±9.50	↓ *	61.28±10.95	39.17±16.14	↓	23.49±9.00	↓ *	70.17±16.18	↑	77.74±19.67	↑
Proteobacteria	34.55±15.59	↑	19.56±6.39	28.40±13.74	↑	47.12±20.27	↑ *	9.32±2.06	↓ *	5.14±0.89	↓ *
Bacteroidetes	6.34±2.73	↑	5.23±1.60	6.65±2.98	↑	5.25±4.97	↑	16.17±6.86	↑ *	7.24±2.17	↑
Actinobacteria	5.45±4.08	↓	12.04±8.01	5.62±0.77	↓	3.85±1.83	↓	3.53±1.55	↓	9.54±3.52	↓
Cyanobacteria	7.64±4.04	↑ *	0.12±0.05	6.26±2.21	↑ *	4.48±1.51	↑ *	0.06±0.28	↓	0.02±0.00	↓ *
Thaumarchaeota	5.01±2.31	↑ *	0.61±0.30	4.43±2.98	↑	3.29±1.20	↑ *	0.24±0.11	↓	0.11±0.07	↓ *
Thermotogae	1.76±1.08	↑ *	0.01±0.01	1.55±1.06	↑	4.68±2.88	↑ *	0	↓	0	↓

[a] Compared with the HSHF group
