# Supplementary material for: Modulation of the Gut Microbiota by Krill Oil in Mice Fed a High-Sugar High-Fat Diet
Source: Front Microbiol. 2017 May 17;8:905. doi: 10.3389/fmicb.2017.00905 (PMC5434167; doi:10.3389/fmicb.2017.00905)
Supplement: Table S5 — The RDP classifications of the sequence reads at the class level. Data are presented as the means ± S.D. Differences were assessed by ANOVA. *P < 0.05, compared with the HSHF group. [file Table5.PDF]

Table S5. The RDP classifications of the sequence reads at the class level. Data are presented as the means ± S.D. **P*<0.05, compared with the HSHF group.

**CLASS**	**Control**		**HSHF**	**HSHF+LD**		**HSHF+MD**		**HSHF+HD**		**HSHF+S**	
	Ratio (%)	Change [a]	Ratio (%)	Ratio (%)	Change [a]	Ratio (%)	Change [a]	Ratio (%)	Change [a]	Ratio (%)	Change [a]
Bacilli	16.13±3.74	↑	15.87±9.01	25.46±10.08	↑	10.56±3.88	↓	58.81±10.06	↑ *	14.94±3.91	↓
Erysipelotrichia	1.00±0.63	↓ *	37.28±17.11	1.73±1.12	↓ *	0.39±0.17	↓ *	3.26±1.98	↓ *	55.63±13.64	↑
Gammaproteobacteria	13.78±6.32	↑ *	3.25±1.09	11.88±3.97	↑ *	32.97±15.99	↑ *	2.59±1.01	↓	0.74±0.09	↓ *
Clostridia	12.23±4.82	↑	7.90±5.05	11.07±6.19	↑	11.85±7.51	↑	7.99±4.01	↑	7.14±3.12	↓
Betaproteobacteria	11.14±5.54	↑	8.08±3.87	8.90±2.39	↑	8.02±3.19	↑	4.79±2.06	↓	2.43±0.98	↓
Actinobacteria	5.43±2.12	↓	12.04± 3.98	5.61±1.29	↓	3.84±0.99	↓ *	3.53±1.11	↓ *	9.54±3.32	↓
Bacteroidia	2.55±0.98	↓	4.97± 2.77	3.54±1.03	↓	2.32±1.08	↓	16.02±4.92	↑ *	7.18±3.09	↑
Alphaproteobacteria	8.49±3.11	↑ *	2.49± 1.27	6.39±3.34	↑	5.30±2.97	↑	1.08±0.17	↓	0.54±0.06	↓
unclassified	7.04±3.51	↑ *	0.85± 0.32	6.42±2.98	↑ *	5.60±4.01	↑	0.35±0.13	↓	0.18±0.12	↓ *
Chloroplast	7.63±4.01	↑ *	0.11± 0.04	6.21±2.09	↑ *	4.45±1.94	↑ *	0.06±0.03	↓	0.02±0.00	↓ *
Deltaproteobacteria	0.91±0.09	↓ *	5.63± 2.83	1.02±0.45	↓ *	0.68±0.60	↓ *	0.32±0.18	↓ *	1.32±0.66	↓
Thermotogae	1.76±0.53	↑ *	0.01± 0.00	1.55±1.00	↑	4.68±2.91	↑ *	0	↓	0	↓

[a] Compared with the HSHF group
