# Supplementary material for: Modulation of the Gut Microbiota by Krill Oil in Mice Fed a High-Sugar High-Fat Diet
Source: Front Microbiol. 2017 May 17;8:905. doi: 10.3389/fmicb.2017.00905 (PMC5434167; doi:10.3389/fmicb.2017.00905)
Supplement: Table S6 — Seven abundant genera in all groups. Data are presented as the means ± S.D. *P < 0.05, compared with the HSHF group. [file Table6.PDF]

Table S6. Seven abundant genera in all groups. Data are presented as the means ± S.D. *P<0.05, compared with the HSHF group.

Phylum	Genus	Control (%) [a]	HSHF (%)	HSHF+LD (%) [a]	HSHF+MD (%) [a]	HSHF+HD (%) [a]	HSHF+S[a]
	Unclassified	7.15±2.76 ↑	2.56±1.23	6.65±0.80 ↑ *	6.00±2.94 ↑	3.10±1.32 ↑	3.64±2.21 ↑
Firmicutes	*Bacillus*	2.68±0.49 ↑	2.65±1.47	2.37±1.28 ↓	2.02±0.83 ↓	5.24±2.74 ↑	1.97±0.98 ↓
Firmicutes	*Lactobacillus*	5.1±2.40 ↑	4.14±2.36	11.53±4.64 ↑	12.22±6.30 ↑	2.60±1.05 ↓	2.92±1.03 ↓
Firmicutes	*Streptococcus*	2.66±1.01 ↑ *	1.01±0.08	2.73±1.59 ↑	1.83±1.01 ↑	0.56±0.29 ↓	0.23±0.05 ↓ *
Firmicutes	*Staphylococcus*	1.26±0.45 ↑	0.50±0.18	5.19±3.63 ↑	2.93±1.73 ↑	37.03±16.72 ↑ *	0.43±0.13 ↓
Proteobacteria	*Delftia*	4.19±0.89 ↑ *	1.99±0.25	3.9±1.30 ↑	3.48±1.92 ↑	1.68±0.78 ↓	0.35±0.31 ↓ *
Proteobacteria	*Ralstonia*	1.46±0.70 ↓	5.24±3.21	0.44±0.28 ↓	0.85±0.31 ↓	1.93±1.11 ↓	1.13±0.65 ↓
Proteobacteria	*Serratia*	4.62±2.25 ↑	1.36±1.00	4.10±1.73 ↑	3.48±2.41 ↑	0.99±0.16 ↓	0.25±0.26 ↓

[a] Compared with the HSHF group
