# Supplementary material for: Modulation of the Gut Microbiota by Krill Oil in Mice Fed a High-Sugar High-Fat Diet
Source: Front Microbiol. 2017 May 17;8:905. doi: 10.3389/fmicb.2017.00905 (PMC5434167; doi:10.3389/fmicb.2017.00905)
Supplement: Table S7 — Sixty-five key OTUs respond to krill oil treatment as identified by redundancy analysis (RDA). [file Table7.PDF]

**Table S7. Sixty-five key OTUs respond to krill oil treatment as identified by redundancy analysis (RDA).**

| OTU name | Phylum | Class | Order | Family | Genus |
|---|---|---|---|---|---|
| OTU00455 | Acidobacteria | *Acidobacteria* | | | *Gp4* |
| OTU00163 | Actinobacteria | *Actinobacteria* | *Actinomycetales* | *Cellulomonadaceae* | *Actinotalea* |
| OTU00172 | Bacteroidetes | *Sphingobacteriia* | *Sphingobacteriales* | *Chitinophagaceae* | *Ferruginibacter* |
| OTU00601 | Bacteroidetes | *Flavobacteriia* | *Flavobacteriales* | *Flavobacteriaceae* | *Cloacibacterium* |
| OTU00215 | Bacteroidetes | *Bacteroidia* | *Bacteroidales* | *Porphyromonadaceae* | *Macellibacteroides* |
| OTU02035 | Euryarchaeota | *Thermoplasmata* | *Methanomassiliicoccales* | *Methanomassiliicoccaceae* | *Methanomassiliicoccus* |
| OTU06982 | Firmicutes | *Bacilli* | *Lactobacillales* | *Carnobacteriaceae* | *Granulicatella* |
| OTU00119 | Firmicutes | *Clostridia* | *Clostridiales* | *Clostridiales_Incertae Sedis XII* | *Fusibacter* |
| OTU00005 | Firmicutes | *Erysipelotrichia* | *Erysipelotrichales* | *Erysipelotrichaceae* | *Allobaculum* |
| OTU00080 | Firmicutes | *Erysipelotrichia* | *Erysipelotrichales* | *Erysipelotrichaceae* | *Allobaculum* |
| OTU06976 | Firmicutes | *Erysipelotrichia* | *Erysipelotrichales* | *Erysipelotrichaceae* | *Coprobacillus* |
| OTU00270 | Firmicutes | *Clostridia* | *Clostridiales* | *Ruminococcaceae* | *unclassified* |
| OTU00001 | Firmicutes | *Bacilli* | *Bacillales* | *Staphylococcaceae* | *Staphylococcus* |
| OTU05500 | Firmicutes | *Negativicutes* | *Selenomonadales* | *Veillonellaceae* | *Anaeroarcus* |
| OTU02168 | Fusobacteria | *Fusobacteriia* | *Fusobacteriales* | *Leptotrichiaceae* | *Leptotrichia* |
| OTU10293 | Planctomycetes | *Planctomycetia* | *Planctomycetales* | *Planctomycetaceae* | *Rubinisphaera* |
| OTU00321 | Proteobacteria | *Alphaproteobacteria* | *Caulobacterales* | *Caulobacteraceae* | *Brevundimonas* |
| OTU00074 | Proteobacteria | *Gammaproteobacteria* | *Enterobacteriales* | *Enterobacteriaceae* | *Enterobacter* |
| OTU00219 | Proteobacteria | *Gammaproteobacteria* | *Enterobacteriales* | *Enterobacteriaceae* | *Enterobacter* |
| OTU01117 | Proteobacteria | *Gammaproteobacteria* | *Enterobacteriales* | *Enterobacteriaceae* | *unclassified* |
| OTU03840 | Proteobacteria | *Gammaproteobacteria* | *Pasteurellales* | *Pasteurellaceae* | *Haemophilus* |
| OTU00054 | Proteobacteria | *Gammaproteobacteria* | *Pseudomonadales* | *Pseudomonadaceae* | *Pseudomonas* |
| OTU00363 | Proteobacteria | *Gammaproteobacteria* | *Pseudomonadales* | *Pseudomonadaceae* | *Pseudomonas* |
| OTU00194 | Proteobacteria | *Alphaproteobacteria* | *Rhizobiales* | *Rhizobiaceae* | *Rhizobium* |
| OTU00187 | Proteobacteria | *Alphaproteobacteria* | *Rhizobiales* | *Rhizobiaceae* | *Shinella* |
| OTU00089 | Proteobacteria | *Gammaproteobacteria* | *Vibrionales* | *Vibrionaceae* | *Vibrio* |
| OTU00015 | Actinobacteria | *Actinobacteria* | *Bifidobacteriales* | *Bifidobacteriaceae* | *Bifidobacterium* |

| | | | | | |
|---|---|---|---|---|---|
| **OTU00030** | Actinobacteria | *Actinobacteria* | *Bifidobacteriales* | *Bifidobacteriaceae* | *Bifidobacterium* |
| **OTU01686** | Actinobacteria | *Actinobacteria* | *Bifidobacteriales* | *Bifidobacteriaceae* | *Bifidobacterium* |
| **OTU00028** | Actinobacteria | *Actinobacteria* | *Coriobacteriales* | *Coriobacteriaceae* | *Enterorhabdus* |
| **OTU00037** | Actinobacteria | *Actinobacteria* | *Coriobacteriales* | *Coriobacteriaceae* | *Olsenella* |
| **OTU00085** | Actinobacteria | *Actinobacteria* | *Coriobacteriales* | *Coriobacteriaceae* | *Paraeggerthella* |
| **OTU00035** | Actinobacteria | *Actinobacteria* | *Coriobacteriales* | *Coriobacteriaceae* | *unclassified* |
| **OTU00327** | Actinobacteria | *Actinobacteria* | *Actinomycetales* | *Micrococcaceae* | *Arthrobacter* |
| **OTU00492** | Bacteroidetes | *Sphingobacteriia* | *Sphingobacteriales* | *Chitinophagaceae* | *Sediminibacterium* |
| **OTU00368** | Bacteroidetes | *Flavobacteriia* | *Flavobacteriales* | *Flavobacteriaceae* | *Chryseobacterium* |
| **OTU07128** | Bacteroidetes | *Bacteroidia* | *Bacteroidales* | *Porphyromonadaceae* | *Barnesiella* |
| **OTU09275** | Bacteroidetes | *Bacteroidia* | *Bacteroidales* | *Porphyromonadaceae* | *Barnesiella* |
| **OTU05459** | Chloroflexi | *Anaerolineae* | *Anaerolineales* | *Anaerolineaceae* | *Bellilinea* |
| **OTU03813** | Chloroflexi | *Anaerolineae* | *Anaerolineales* | *Anaerolineaceae* | *Longilinea* |
| **OTU02243** | Firmicutes | *Bacilli* | *Lactobacillales* | *Carnobacteriaceae* | *Carnobacterium* |
| **OTU00002** | Firmicutes | *Erysipelotrichia* | *Erysipelotrichales* | *Erysipelotrichaceae* | *Allobaculum* |
| **OTU00548** | Firmicutes | *Erysipelotrichia* | *Erysipelotrichales* | *Erysipelotrichaceae* | *Erysipelotrichaceae* |
| **OTU00498** | Firmicutes | *Clostridia* | *Clostridiales* | *Lachnospiraceae* | *Acetatifactor* |
| **OTU00019** | Firmicutes | *Clostridia* | *Clostridiales* | *Lachnospiraceae* | *Clostridium XlVa* |
| **OTU00108** | Firmicutes | *Clostridia* | *Clostridiales* | *Lachnospiraceae* | *Clostridium XlVa* |
| **OTU00155** | Firmicutes | *Clostridia* | *Clostridiales* | *Lachnospiraceae* | *Clostridium XlVa* |
| **OTU00310** | Firmicutes | *Clostridia* | *Clostridiales* | *Lachnospiraceae* | *Clostridium XlVa* |
| **OTU03792** | Firmicutes | *Clostridia* | *Clostridiales* | *Lachnospiraceae* | *Clostridium XlVa* |
| **OTU08630** | Firmicutes | *Clostridia* | *Clostridiales* | *Lachnospiraceae* | *Roseburia* |
| **OTU00280** | Firmicutes | *Clostridia* | *Clostridiales* | *Lachnospiraceae* | *unclassified* |
| **OTU00007** | Firmicutes | *Bacilli* | *Lactobacillales* | *Lactobacillaceae* | *Lactobacillus* |
| **OTU00009** | Firmicutes | *Bacilli* | *Lactobacillales* | *Lactobacillaceae* | *Lactobacillus* |
| **OTU00027** | Firmicutes | *Clostridia* | *Clostridiales* | *Peptostreptococcaceae* | *Romboutsia* |
| **OTU00359** | Firmicutes | *Bacilli* | *Bacillales* | *Planococcaceae* | *Solibacillus* |
| **OTU11697** | Firmicutes | *Bacilli* | *Lactobacillales* | *Streptococcaceae* | *Streptococcus* |
| **OTU00316** | Fusobacteria | *Fusobacteriia* | *Fusobacteriales* | *Leptotrichiaceae* | *Leptotrichia* |
| **OTU02033** | Proteobacteria | *Deltaproteobacteria* | *Bdellovibrionales* | *Bdellovibrionaceae* | *Vampirovibrio* |
| **OTU00014** | Proteobacteria | *Betaproteobacteria* | *Burkholderiales* | *Burkholderiaceae* | *Ralstonia* |
| **OTU00033** | Proteobacteria | *Alphaproteobacteria* | *Caulobacterales* | *Caulobacteraceae* | *Caulobacter* |

| | | | | | |
|---|---|---|---|---|---|
| **OTU05657** | Proteobacteria | *Betaproteobacteria* | *Burkholderiales* | *Comamonadaceae* | *Diaphorobacter* |
| **OTU00004** | Proteobacteria | *Deltaproteobacteria* | *Desulfovibrionales* | *Desulfovibrionaceae* | *Desulfovibrio* |
| **OTU00525** | Proteobacteria | *Epsilonproteobacteria* | *Campylobacterales* | *Helicobacteraceae* | *Sulfurovum* |
| **OTU00221** | Proteobacteria | *Gammaproteobacteria* | *Xanthomonadales* | *Sinobacteraceae* | *Nevskia* |
| **OTU00307** | Proteobacteria | *Alphaproteobacteria* | *Sphingomonadales* | *Sphingomonadaceae* | *Sphingomonas* |
| **OTU00015** | Actinobacteria | *Actinobacteria* | *Bifidobacteriales* | *Bifidobacteriaceae* | *Bifidobacterium* |
| **OTU00030** | Actinobacteria | *Actinobacteria* | *Bifidobacteriales* | *Bifidobacteriaceae* | *Bifidobacterium* |
| **OTU01686** | Actinobacteria | *Actinobacteria* | *Bifidobacteriales* | *Bifidobacteriaceae* | *Bifidobacterium* |
| **OTU00028** | Actinobacteria | *Actinobacteria* | *Coriobacteriales* | *Coriobacteriaceae* | *Enterorhabdus* |
| **OTU00037** | Actinobacteria | *Actinobacteria* | *Coriobacteriales* | *Coriobacteriaceae* | *Olsenella* |
| **OTU00085** | Actinobacteria | *Actinobacteria* | *Coriobacteriales* | *Coriobacteriaceae* | *Paraeggerthella* |
| **OTU00035** | Actinobacteria | *Actinobacteria* | *Coriobacteriales* | *Coriobacteriaceae* | *unclassified* |
| **OTU00327** | Actinobacteria | *Actinobacteria* | *Actinomycetales* | *Micrococcaceae* | *Arthrobacter* |
| **OTU00492** | Bacteroidetes | *Sphingobacteriia* | *Sphingobacteriales* | *Chitinophagaceae* | *Sediminibacterium* |

Red: OTUs with enhanced abundance compared with the HSHF group.

Green: OTUs with reduced abundance compared with the HSHF group.
