# Supplementary material for: Modulation of the Gut Microbiota by Krill Oil in Mice Fed a High-Sugar High-Fat Diet
Source: Front Microbiol. 2017 May 17;8:905. doi: 10.3389/fmicb.2017.00905 (PMC5434167; doi:10.3389/fmicb.2017.00905)
Supplement: Table S8 — Relative average abundance of the 65 key OTUs responding to krill oil treatment as identified by redundancy analysis (RDA). [file Table8.PDF]

Table S8. Relative average abundance of the 65 key OTUs responding to krill oil treatment as identified by redundancy analysis (RDA).

OTU name	Control (%)	HSHF (%)	HSHF+LD (%)	HSHF+MD (%)	HSHF+HD (%)
OTU00455	0.008185986	0.003795354	0.021355657	0.032405457	0.00420221
OTU00163	0.130975769	0.003795354	0.025626789	0.042127094	0.00420221
OTU00172	0.10232482	0.007590709	0.098236023	0.071292006	0.008404421
OTU00601	0.085952849	0.007590709	0.05979584	0.029164911	0.008404421
OTU00215	0.085952849	0.018976772	0.106778286	0.110178554	0.029415473
OTU02035	0.122789784	0.003795354	0.068338103	0.071292006	0.00420221
OTU06982	0.008185986	0.003795354	0.046982446	0.006481091	0.008404421
OTU00119	0.188277669	0.003795354	0.196472045	0.139343465	0.00420221
OTU00005	0.065487885	0.003795354	0.303250331	0.055089277	0.00420221
OTU00080	0.024557957	0.011386063	0.264810148	0.061570368	0.596713871
OTU06976	0.004092993	0.003795354	0.004271131	0.016202729	0.037819893
OTU00270	0.07367387	0.015181418	0.08969376	0.032405457	0.021011052
OTU00001	1.060085134	0.311219068	4.08320164	2.112835802	30.8190108
OTU05500	0.008185986	0.003795354	0.004271131	0.012962183	0.008404421
OTU02168	0.02865095	0.003795354	0.012813394	0.02268382	0.00420221
OTU10293	0.004092993	0.003795354	0.017084526	0.006481091	0.00420221
OTU00321	0.106417813	0.007590709	0.017084526	0.051848731	0.016808841
OTU00074	0.78994761	0.216335206	0.469824456	0.379143848	0.218514939
OTU00219	0.008185986	0.007590709	0.076880366	0.217116562	0.012606631
OTU01117	0.065487885	0.003795354	0.111049417	0.061570368	0.00420221
OTU03840	0.045022921	0.018976772	0.025626789	0.029164911	0.029415473
OTU00054	0.368369352	0.003795354	0.405757485	0.362941119	0.184897256
OTU00363	0.036836935	0.011386063	0.042711314	0.042127094	0.012606631
OTU00194	0.135068762	0.003795354	0.046982446	0.093975826	0.00420221
OTU00187	0.139161755	0.007590709	0.055524708	0.139343465	0.025213262
OTU00089	0.55664702	0.003795354	0.375859565	0.327295116	0.012606631
OTU00015	0.159626719	7.063154699	0.230641097	0.06805146	0.512669664
OTU00030	0.331532417	0.62623349	0.482637851	0.087494734	0.197503887

**OTU01686**	0.024557957	0.091088508	0.025626789	ND	0.008404421
**OTU00028**	0.008185986	0.265674814	0.025626789	ND	0.016808841
**OTU00037**	0.016371971	1.066494611	0.021355657	0.04536764	0.705971341
**OTU00085**	0.004092993	0.201153788	0.004271131	ND	0.025213262
**OTU00035**	0.040929928	0.33019584	0.046982446	0.019443274	0.075639787
**OTU00327**	0.024557957	0.060725672	0.055524708	0.012962183	0.046224314
**OTU00492**	0.012278978	0.022772127	0.004271131	0.009721637	0.00420221
**OTU00368**	0.02865095	0.03415819	0.025626789	0.016202729	0.016808841
**OTU07128**	0.004092993	0.037953545	0.017084526	0.003240546	0.025213262
**OTU09275**	0.004092993	0.03415819	0.004271131	0.003240546	0.012606631
**OTU05459**	0.020464964	0.026567481	0.017084526	0.025924366	0.00420221
**OTU03813**	0.02865095	0.03415819	0.025626789	0.016202729	0.00420221
**OTU02243**	0.008185986	0.015181418	ND	0.006481091	0.00420221
**OTU00002**	0.568925999	35.34993168	0.226369965	0.035646003	1.97083666
**OTU00548**	ND	0.015181418	0.004271131	0.006481091	0.00420221
**OTU00498**	0.004092993	0.026567481	0.012813394	0.019443274	0.016808841
**OTU00019**	0.004092993	0.573098527	0.115320548	0.038886548	0.100853049
**OTU00108**	ND	0.053134963	0.008542263	0.019443274	0.042022104
**OTU00155**	ND	0.064521026	0.021355657	0.006481091	0.008404421
**OTU00310**	0.012278978	0.030362836	0.02989792	ND	0.025213262
**OTU03792**	0.004092993	0.056930317	0.004271131	ND	0.016808841
**OTU08630**	0.020464964	0.026567481	0.025626789	ND	0.012606631
**OTU00280**	0.004092993	0.11006528	0.008542263	ND	0.058830945
**OTU00007**	1.297478716	5.601943221	3.177721778	0.139343465	0.168088415
**OTU00009**	0.462508186	0.474419311	0.469824456	0.20415438	0.11766189
**OTU00027**	0.613948919	0.675573099	0.525349165	0.401827668	0.273143674
**OTU00359**	0.02865095	0.041748899	0.004271131	0.016202729	0.037819893
**OTU11697**	ND	0.011386063	0.004271131	0.003240546	0.00420221
**OTU00316**	0.032743942	0.03415819	0.025626789	0.019443274	ND
**OTU02033**	ND	0.053134963	0.004271131	0.003240546	0.025213262
**OTU00014**	1.453012443	5.229998482	0.444197668	0.842541884	1.928814556
**OTU00033**	0.012278978	1.366327615	0.034169051	0.074532551	0.529478506
**OTU05657**	0.004092993	0.056930317	ND	0.009721637	0.021011052

**OTU00004**	0.004092993	5.347654471	0.367317302	0.074532551	0.096650838
**OTU00525**	0.008185986	0.030362836	0.012813394	0.009721637	0.025213262
**OTU00221**	0.020464964	0.11006528	0.004271131	ND	0.033617683
**OTU00307**	0.07367387	0.239107333	0.145218468	0.035646003	0.054628735
**OTU00015**	0.008185986	0.003795354	0.021355657	0.032405457	0.00420221
**OTU00030**	0.130975769	0.003795354	0.025626789	0.042127094	0.00420221
**OTU01686**	0.10232482	0.007590709	0.098236023	0.071292006	0.008404421
**OTU00028**	0.085952849	0.007590709	0.05979584	0.029164911	0.008404421
**OTU00037**	0.085952849	0.018976772	0.106778286	0.110178554	0.029415473
**OTU00085**	0.122789784	0.003795354	0.068338103	0.071292006	0.00420221
**OTU00035**	0.008185986	0.003795354	0.046982446	0.006481091	0.008404421
**OTU00327**	0.188277669	0.003795354	0.196472045	0.139343465	0.00420221
**OTU00492**	0.065487885	0.003795354	0.303250331	0.055089277	0.00420221

Red: OTUs with enhanced abundance compared with the HSHF group.

Green: OTUs with reduced abundance compared with the HSHF group.
