# Supplementary figures and images for: Modulation of the Gut Microbiota by Krill Oil in Mice Fed a High-Sugar High-Fat Diet
Source: Front Microbiol. 2017 May 17;8:905. doi: 10.3389/fmicb.2017.00905 (PMC5434167; doi:10.3389/fmicb.2017.00905)

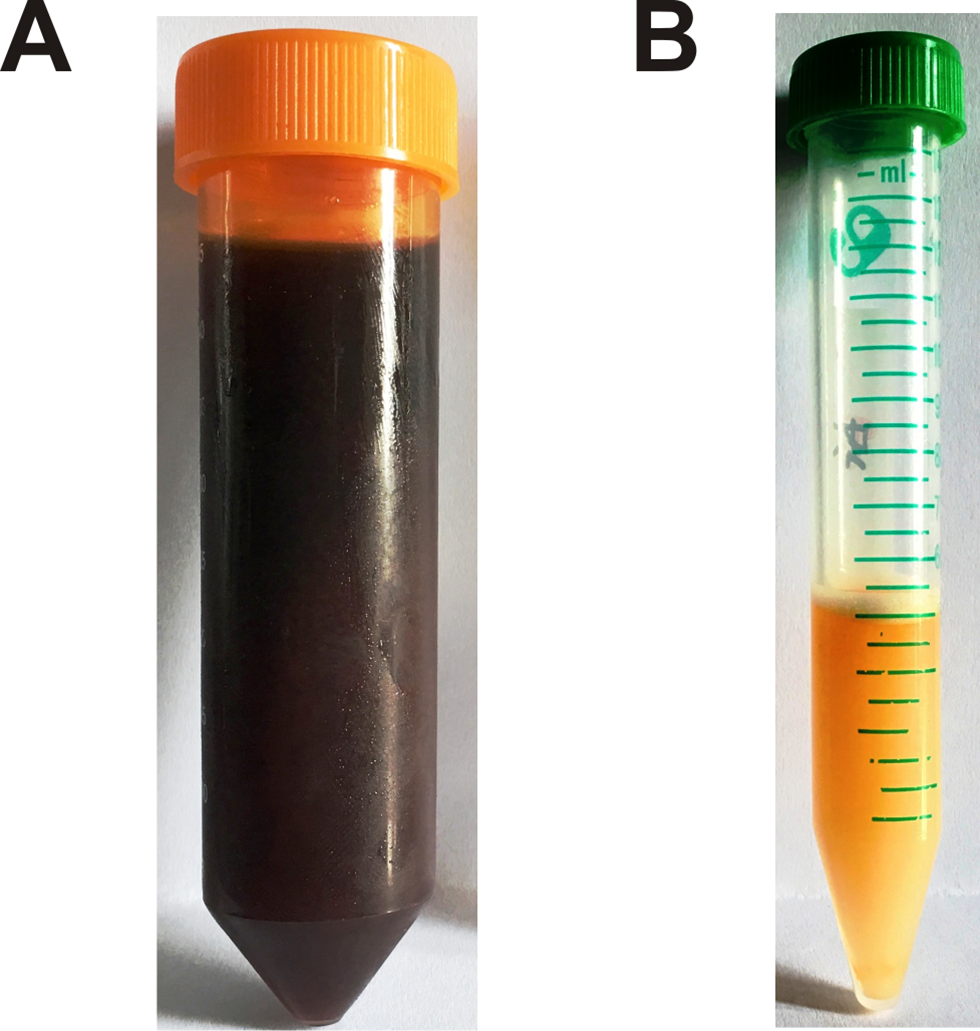

Supplement: Figure S1 — Features of the krill oil. (A) Concentrated krill oil. (B) Diluted krill oil. [file Image1.TIF]

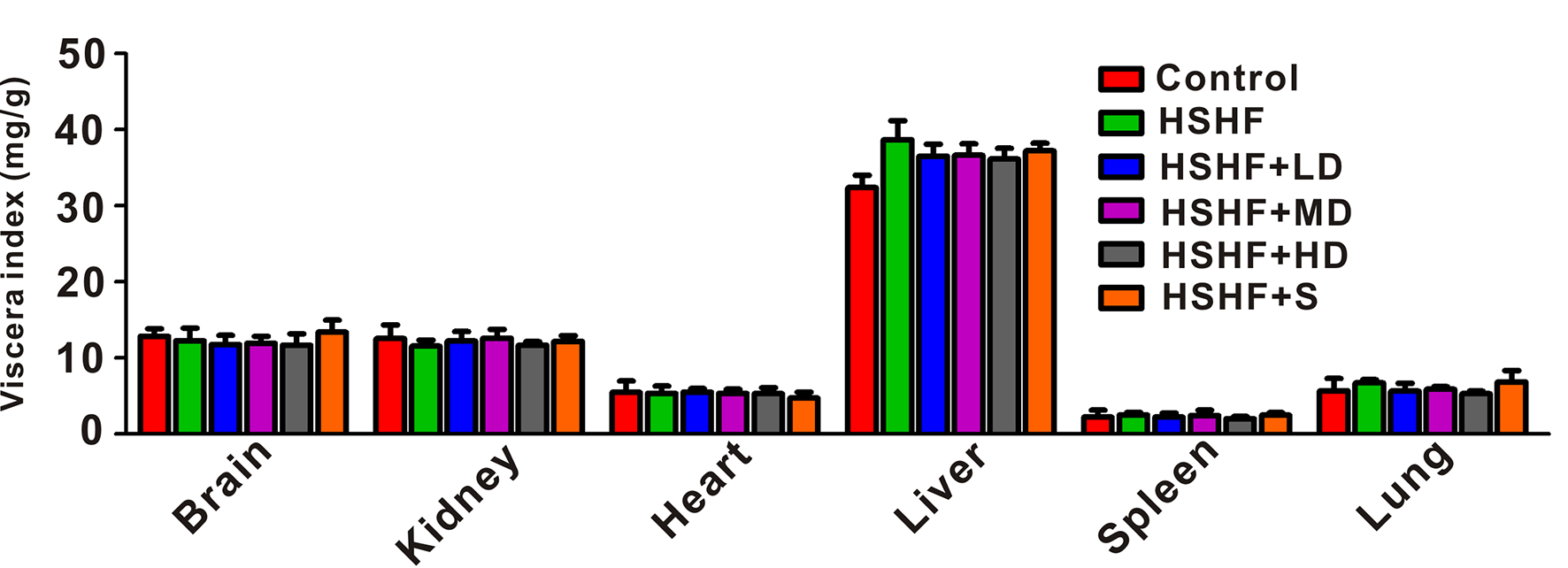

Supplement: Figure S2 — Viscera indices of the mice fed the HSHF diet with krill oil supplementation. Data are presented as the means ± S.D. [file Image2.TIF]

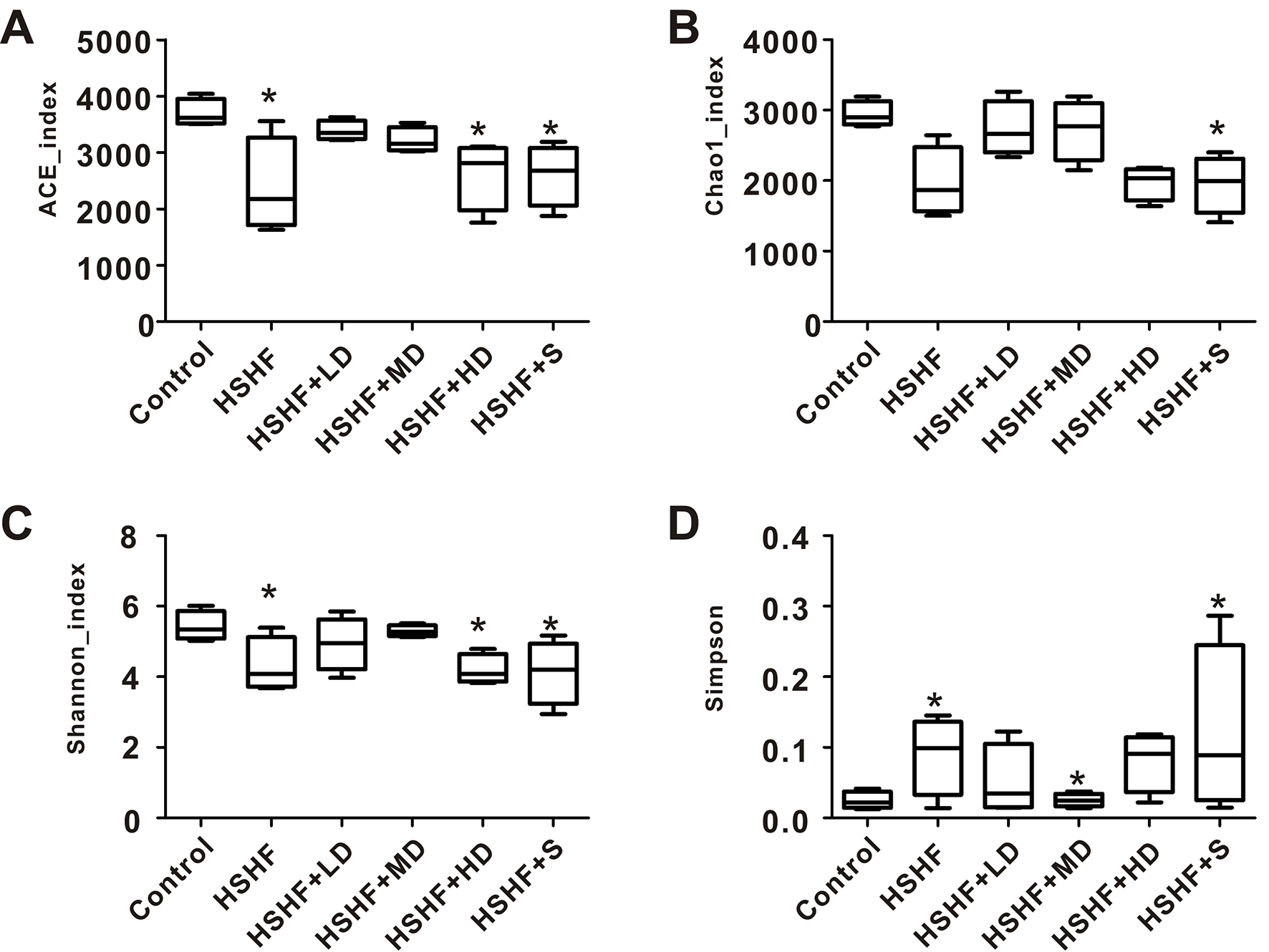

Supplement: Figure S3 — Different microbial diversity indices in different groups. (A) ACE index. (B) Chao1 index. (C) Shannon index. (D) Simpson. *P < 0.05 vs. control group. [file Image3.TIF]

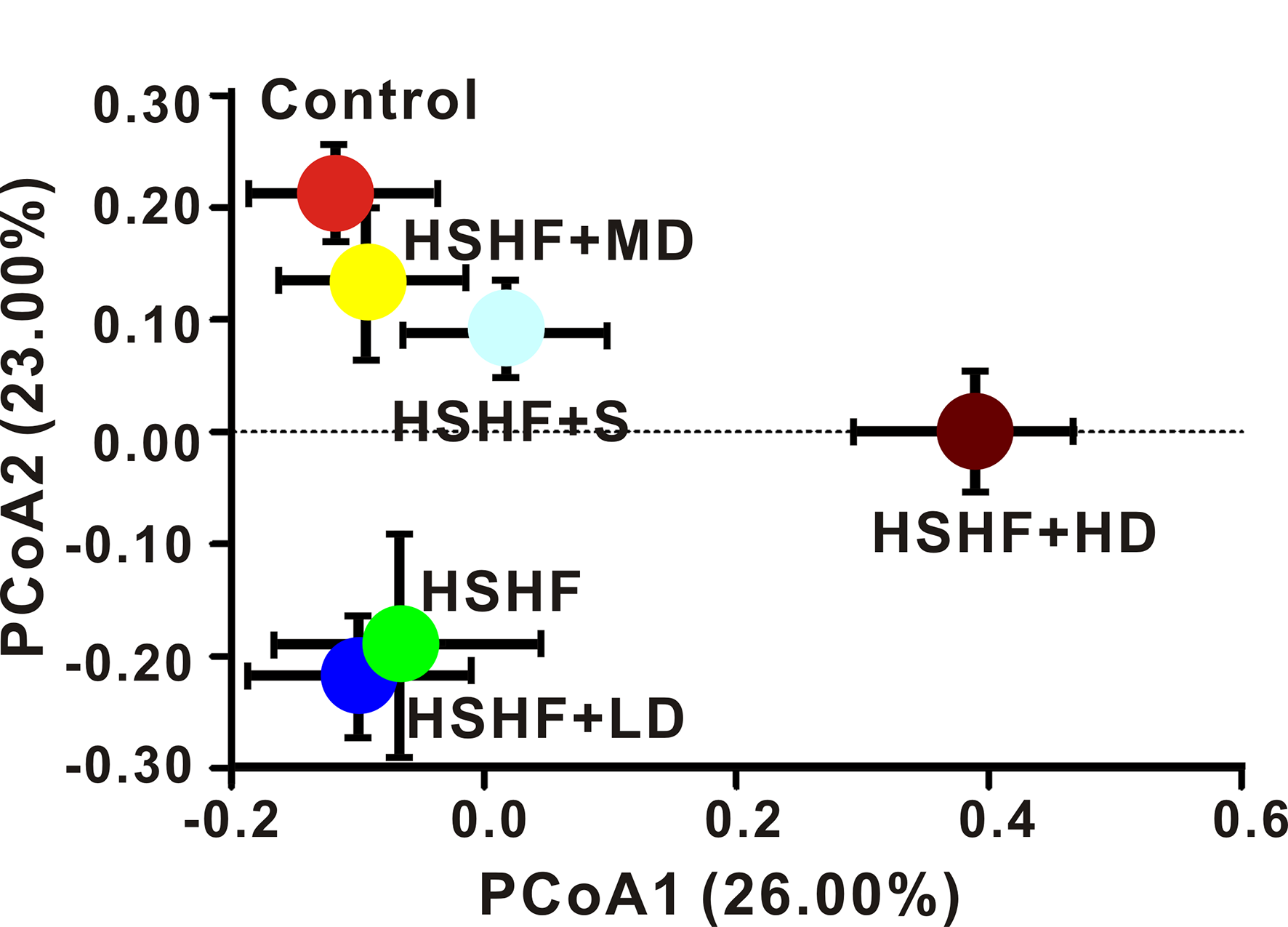

Supplement: Figure S4 — Dose-dependent change in the composition of mice fed the HSHF diet and treated with different krill oil doses via unweighted Unifrac PCoA analysis. Data are presented as the means ± S.D. [file Image4.TIF]
