# Supplementary material for: Modulation of the Gut Microbiota by Krill Oil in Mice Fed a High-Sugar High-Fat Diet
Source: Front Microbiol. 2017 May 17;8:905. doi: 10.3389/fmicb.2017.00905 (PMC5434167; doi:10.3389/fmicb.2017.00905)
Supplement: Supplementary file 13 [file Presentation1.PDF]

Supplementary Material

Modulation of gut microbiota by krill oil during the treatment of high-sugar high-fat diet induced hyperlipidemia in mice

Chenyang Lu, Tingting Sun, Yanyan Li, Dijun Zhang, Jun Zhou, Xiurong Su*

*** Correspondence:** Xiurong Su: suxiurong_public@163.com

Supplementary materials legends

Table S1. The fatty acid components of the krill oil

Table S2. The body weight of mice fed experimental diets. Data are presented as the means ± S.D.

Table S3. Serum lipid indices of mice fed experimental diets. Data are presented as the means ± S.D. *P<0.05, **P<0.01, ***P<0.001, compared with the HSHF group.

Table S4. The RDP classifications of the sequence reads at the phylum level. Data are presented as the means ± S.D. *P<0.05, compared with the HSHF group.

Table S5. The RDP classifications of the sequence reads at the class level. Data are presented as the means ± S.D. *P<0.05, compared with the HSHF group.

Table S6. Seven abundant genera in all groups. Data are presented as the means ± S.D. *P<0.05, compared with the HSHF group.

Table S7. Sixty-five key OTUs respond to krill oil treatment as identified by redundancy analysis (RDA).

Table S8. Relative average abundance of the 65 key OTUs responding to krill oil treatment as identified by redundancy analysis (RDA).

Figure S1 Features of the krill oil. (A) Concentrated krill oil. (B) Diluted krill oil.

Figure S2 Viscera indices of the mice fed the HSHF diet with krill oil supplementation. Data are presented as the means ± S.D.

Figure S3 The numbers of OTUs identified at several different similarity levels.

Figure S4 Different microbial diversity indices in different groups. (A) ACE index. (B) Chao1 index. (C) Shannon index. (D) Simpson.

Figure S5 Dose-dependent change in the composition of mice fed the HSHF diet and treated with different krill oil doses via unweighted Unifrac PCoA analysis. Data are presented as the means ± S.D.
